# Ethical challenges in the bioanthropological and biomedical investigation of Sicilian mummies—Past experience and future pathways

**DOI:** 10.1002/ajpa.24946

**Published:** 2024-05-09

**Authors:** Dario Piombino‐Mascali, Kirsty Squires, Albert Zink

**Affiliations:** ^1^ Institute of Biomedical Sciences, Vilnius University Vilnius Lithuania; ^2^ School of Health, Education, Policing and Sciences Staffordshire University Stoke‐on‐Trent UK; ^3^ Institute for Mummy Studies, EURAC Bolzano Italy

**Keywords:** bioarchaeology, death, dignity, ethics, heritage, mummies

## Abstract

This article presents a multidisciplinary approach adopted in the Sicily mummy project, highlighting unique challenges and major ethical concerns inherent to the scientific study, conservation, and presentation of these mummies. Recognizing mummies as a distinct category of human remains, this paper argues for the development and application of specialized guidelines that address the intricate balance between scientific inquiry and respect for the cultural, religious, and mortuary practices that characterize the cultural context, in this case of Sicily. Through a transparent and collaborative dialogue among all stakeholders—including curators, clergy, scientists, and government officials—the project ensures the preservation of the mummies' dignity within their sacred spaces. The critical role of biological anthropologists is emphasized, alongside the contributions of clinical radiologists, pathologists, and qualified restorers, in constructing a comprehensive understanding of the mummies' biocultural significance. The paper advocates for a bioarchaeological strategy that advances scientific knowledge while safeguarding the mummies and respecting living communities. Additionally, we call for ethical rigor in scholarly publications and suggest future actions to protect this invaluable heritage. This approach not only preserves the dignity and integrity of the mummified remains but also enriches our understanding of past human societies.

## 1 INTRODUCTION

In the study of mummified remains—those deceased humans or animals whose non‐bony tissues, including skin, hair, ligaments, muscles, and even inner organs, are preserved post‐mortem—the variability of preservation reflects both natural environmental conditions and specific anthropogenic treatments aimed at cadaveric preservation (Aufderheide, 2003; Lynnerup, 2007). These remains, once mere curiosities (Baber, 2016), have garnered the scholarly attention necessary for their establishment as crucial resources for biomedical and bioanthropological research (Nystrom et al., 2023; Wilson et al., 2023; Zimmerman, 2012). This shift, marked by the application of scientific methodologies, underscores the evolution of mummy studies into a recognized discipline by 1992 (Cárdenas‐Arroyo & Rodríguez‐Martín, 2021), paralleling the emergence of ethical considerations regarding research methodologies, public display, and the privacy rights of the deceased (Day, 2014; Gill‐Frerking, 2021; Holm, 2001; Kreissl Lonfat et al., 2015; Mytum, 2021; Piombino‐Mascali & Gill‐Frerking, 2019; Squires & Piombino‐Mascali, 2021).

Central to this discourse, the Sicily mummy project, initiated in 2007, embodies an ethical framework in its endeavors to document, study, and conserve the extensive assemblages of preserved human remains in Sicily, an autonomous region of Italy. These assemblages, primarily associated with religious institutions and spanning from the 16th to the 19th centuries—with notable exceptions—underscore the project's hypothesis‐driven methodology, which engages international collaboration across Europe and the United States, focusing on expertise‐driven sub‐projects (Piombino‐Mascali & Zink, 2021; Squires & Piombino‐Mascali, 2024).

Our approach, deeply ingrained with the principle of posthumous dignity, emphasizes non‐destructive, or minimally destructive techniques to mitigate damage to the remains, thereby honoring the essence of these once‐living individuals. This respectful treatment during and after analysis is pivotal (Beckett et al., 2020; Musshoff et al., 2013; Panzer et al., 2010; Panzer et al., 2013; Panzer et al., 2018; Piombino‐Mascali et al., 2015; Piombino‐Mascali et al., 2017; Seiler et al., 2017).

In Italy, protocols and recommendations concerning the research and ethics of human remains have been articulated only recently (CNR, 2019; Riga, 2022), yet the engagement with mummified remains demands a nuanced ethical consideration. This paper presents the Sicily mummy project's ethical framework as a model for future research in Sicily and beyond, highlighting the importance of dignity, respect, and ethical integrity in the study of mummified remains.

## 2 LEGISLATIVE BACKGROUND OF SICILIAN MUMMIES

**FIGURE 1:**
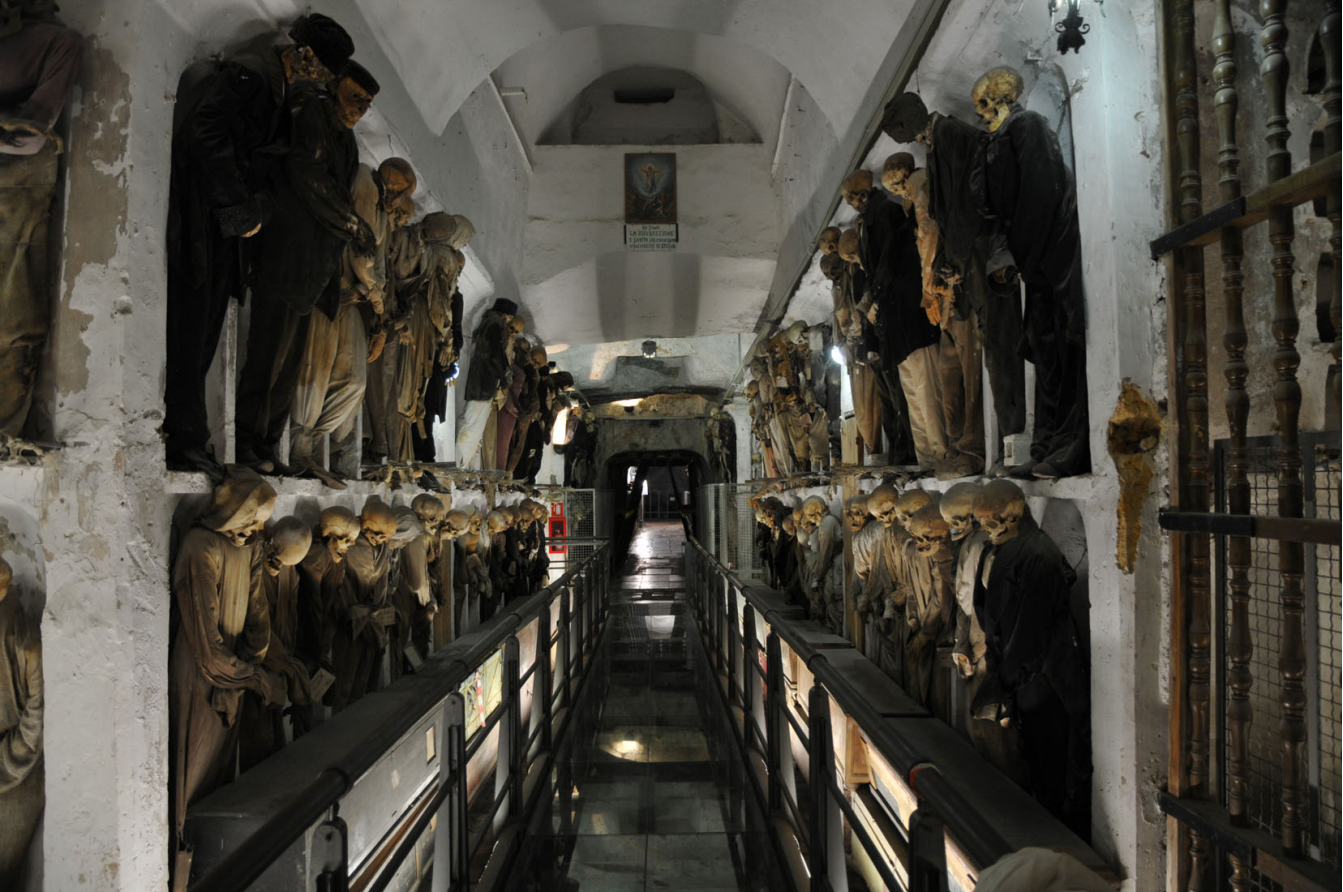
The Capuchin Catacombs of Palermo are the largest Sicilian assemblage of mummified and skeletonized human remains (Image courtesy of the Sicily mummy project, 2010).

The mummies under consideration in this study, nestled within the sacred confines of churches, convents, and chapels across Sicily, are intimately linked to subterranean chambers or annexed rooms, embodying a wide spectrum of communal memory—from the handful of preserved bodies in Novara di Sicilia to the vast congregation of the dead in Palermo's Capuchin Catacombs (Figure 1). These sacred sites, often under the guardianship of Sicily's religious jurisdictions, house the remains, not as commodities, but as *res extra commercium*, entities beyond the reach of commerce, reflecting a deep respect for the deceased (Shevelev & Shevelev, 2023). In instances where the care of these spaces falls to brotherhoods, local councils, or even the state—especially after the 1861 unification acts (Campobello, 2015)—each caretaker upholds their protection under Italy's stringent cultural heritage laws, underscoring the intrinsic value of these remains as touchstones of historical life (Colaianni, 2012; Piombino‐Mascali & Zink, 2011).

This protective stance is further nuanced by Sicily's autonomous governance over its cultural treasures since 1975, entrusting the Department of Cultural Heritage and Sicilian Identity with the oversight of these invaluable assets (Campo, 2008). The department, through its superintendencies for the cultural and environmental heritage, embodies a holistic approach to preservation, extending its care to all manifestations of cultural legacy, including the ethno‐anthropological heritage represented by the mummies. This designation highlights the mummies' significance not merely as historical artifacts but as bearers of the island's rich cultural and traditional tapestry (Piombino‐Mascali & Zink, 2011).

In recognition of the unique nature of these remains, and to honor their dignity, a novel role was established within the department—the honorary inspector to the mummified bio‐anthropological heritage of the Sicilian territory (2011). This position, held by the lead author, who shares a profound cultural and spiritual connection with Sicily and its Catholic heritage, is pivotal in guiding the stewardship of these remains. Through this role, comprehensive guidelines have been developed, addressing the maintenance of the decedents, the conduct of visitors, and the optimal conditions for the preservation of these remains, thereby ensuring their respect and integrity for generations to come (Samadelli et al., 2013).

We, the authors, advocate for the thoughtful conservation and curation of these mummies, recognizing their profound impact on contemporary society. They are not only relics of mortuary customs and cultural identity but also evoke a collective emotional journey through the landscape of human loss. In Italy, the sanctity of these remains is safeguarded under the law, reflecting a commitment to preserving the dignity of the past and the lessons it imparts for the future (Piombino‐Mascali & Zink, 2011).

## 3 WHY ARE THE SICILIAN DEAD DISPLAYED?

Amid the discourse surrounding the exhibition of Sicilian mummies, concerns emerge from both specialists and the general populace, rooted in apprehensions that without a deep understanding of the historical cultural practices and beliefs, the display of these mummies could lead to misinterpretations by visitors and locals alike. Such practices, especially within the Catholic tradition, often trace back to the veneration of early martyrs' relics, showcasing a longstanding tradition of enshrining clergy members' bodies (Fulcheri, 2013). Unique to Sicily and the South of Italy, the individuals interred within these sacred crypts and chapels are not canonized saints or notably distinguished religious figures but chose—or were chosen by their families or religious communities—for a distinct funerary process. This method relied on natural yet enhanced dehydration techniques, eschewing invasive procedures such as incision or organ removal, within specifically designed architectural features aimed at facilitating this process.

The bodies were placed on masonry structures, often within the proximity of exhibition spaces, to allow for the efficient drainage of bodily fluids during decomposition, a technique observed in various locales including Palermo, Savoca, and Piraino (Fornaciari et al., 2010). This careful treatment was available to individuals from diverse social strata, including clergy, nobility, and the bourgeoisie, allowing their physical form to be preserved for extended periods before eventual preparation for public display or coffin burial.

In contrast, a minority of Sicilian mummies underwent anthropogenic preservation methods, involving evisceration and the use of embalming fluids, mirroring the care bestowed upon their spontaneously mummified counterparts (Panzer et al., 2010). This nuanced approach to death and body preservation reflects a profound cultural resonance with the concept of double burials, as theorized by sociologist Robert Hertz, suggesting a symbolic journey of the soul paralleled by the cadaver's transformation (Hertz, 1978). Such rites often concluded with the deceased's exhibition, emblematic of their final resting phase and reinforcing the intertwining of death and communal memory within Sicilian and Southern Italian societies.

Moreover, these practices bear witness to the Christian cult of the purging souls, especially prevalent in the 19th century, where the mummified individuals became protective entities within a reciprocal relationship between the living and the dead (Piombino‐Mascali & Nystrom, 2020). This tradition, where living people engage in regular visits to their deceased kin to solicit protection, health, and fortune, underscores the cultural imperative to continue displaying Sicilian mummies (Guarino, 2022a, 2022b). It is not merely an act of preservation but a vibrant testament to the enduring connections and cultural legacies that define Sicilian identity and its spiritual landscape.

## 4 RECENT DEVELOPMENTS IN THE STUDY AND CARE OF THE SICILIAN DEAD

### 4.1 A non‐destructive or minimally destructive approach

In a departure from traditional methodologies, our research on Sicilian mummies deliberately eschews the practice of full‐body dissection. Historically, mummy autopsies have been a cornerstone of paleopathological research worldwide, and indeed, this method was prevalent in Italy well into the early 21st century (Piombino‐Mascali & Gill‐Frerking, 2019). Traditionally favored by paleopathologists with medical training, the evolution of the discipline towards bioarchaeology has rendered such invasive techniques less common and, in many cases, unnecessary (Nystrom, 2019), save for a few specific instances where they may be justified (Kim et al., 2021).

The irreversible nature of autopsies, coupled with the permanent alterations inflicted on the remains, poses significant limitations for future research endeavors. Moreover, this invasive approach fails to honor the sanctity with which these mummified individuals are regarded. Specifically, in Sicily, autopsies were conducted on mummies from the Capuchin Church of Comiso in 1987, marking a rare instance of such intervention (Fornaciari, 1998). Since the resurgence of interest in Sicilian mummies in 2007, our strategy has shifted towards minimal intervention, reserving sampling for instances of taphonomic changes or when pre‐existing conditions permit access to internal structures without further damage (Piombino‐Mascali et al., 2017).

This methodology aligns with several critical considerations: the preservation of the mummies' heritage status, which precludes visible alterations; respect for the deceased's final wishes for their remains to rest in sacred settings; and adherence to ethical standards that recognize the preservation of bodily integrity as a right that extends beyond death, as affirmed by the Universal Declaration of Human Rights (1948) (Kreissl Lonfat et al., 2015).

Looking ahead, we advocate for the responsible collection of samples—subject to appropriate permissions—which should then be stored in a biobank. This repository would facilitate a variety of analyses, including genetic, isotopic, and histological studies, without necessitating further direct contact with the mummified remains (Lynnerup, 2009). By adopting such practices, we ensure the safeguarding of these invaluable collections for future generations, maintaining their integrity while still unlocking the secrets they hold.

### 4.2 *In situ* imaging

In the pursuit of understanding the paleopathology of these Sicilian mummies, researchers are met with significant obstacles. The mummies' traditional attire and the presence of religious artifacts can obscure potential lesions, while taphonomic processes further complicate matters by altering or destroying internal organs (Aufderheide, 2003). Despite these challenges, advancements in imaging technology, combined with the expertise of a skilled paleoradiologist, enable a non‐invasive exploration of these ancient remains.

**FIGURE 2:**
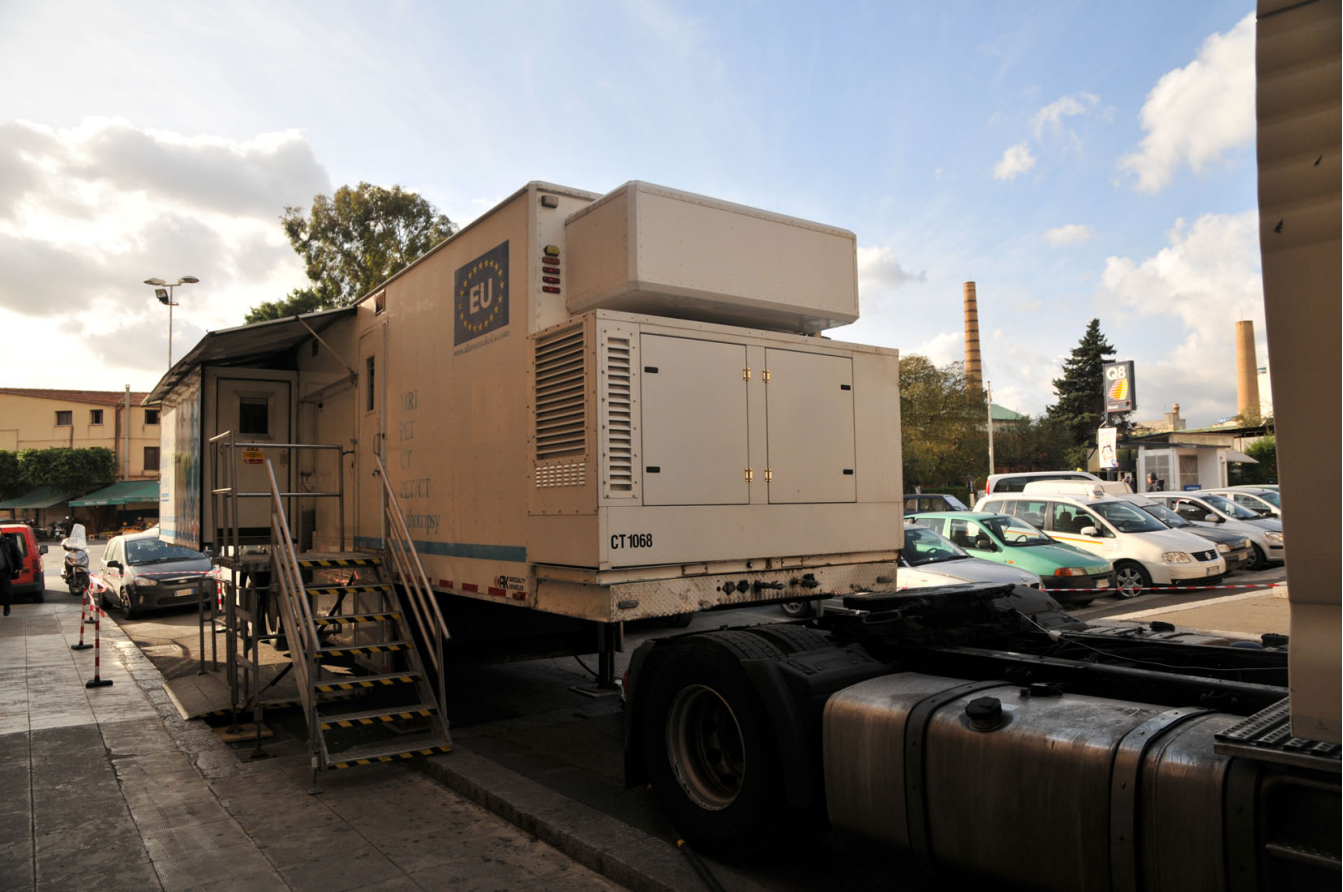
A mobile unit outside the Capuchin Church of Palermo, Sicily (Image courtesy of the Sicily mummy project, 2010).

Our methodology embraces the use of digital radiography and computed tomographic (CT) scanning to penetrate the mummies' shrouded secrets without physical intrusion. CT scanning offers superior image quality and detailed insights into any taphonomic alterations, pathological lesions, or evidence of trauma within the soft tissues that remain (Beckett, 2014). The logistical constraints of transporting these mummies—often due to their fragile state or the sacrosanct spaces they inhabit—have led to the adoption of mobile CT scanning units for our research in Sicily (Figure 2) (Panzer et al., 2018). However, the practicalities of deploying such equipment in the region's quaint, narrow‐streeted towns necessitate a more adaptable solution. Thus, a portable direct digital X‐ray unit emerges as a pragmatic alternative, ensuring that the examination process respects the physical and spiritual integrity of the mummies and addresses the concerns of all stakeholders involved (Beckett et al., 2020; Panzer et al., 2010; Piombino‐Mascali et al., 2015; Piombino‐Mascali et al., 2017).

Moreover, the health and safety implications of interacting with these preserved remains cannot be overlooked (Ventura et al., 2022). The presence of active microorganisms within the crypts and on the mummies themselves presents a not insignificant health risk, not only to the researchers but also potentially to the broader public should these remains be analyzed within healthcare facilities (Piñar et al., 2013). The risk of biodeterioration and the potential for increased microbial activity due to the manipulation of the mummies necessitate stringent precautions to mitigate exposure to harmful biological agents.

Accordingly, any planned analysis must be preceded by a comprehensive risk assessment, followed by the implementation of stringent safety protocols to safeguard against the potential health hazards these ancient remains may pose (DeAraujo et al., 2016; Naji et al., 2014; Piñar et al., 2013; Šimonovičová et al., 2015). This approach is aligned with legal mandates aimed at protecting individuals in their workplaces and underscores the importance of ethical and safety considerations in the study of Sicilian mummies. Through such measures, we ensure that our research is conducted with the utmost respect for both the deceased and the living, honoring our commitment to ethical rigor, and safety in the exploration of Sicily's rich paleopathological heritage (Squires & Piombino‐Mascali, 2024).

### 4.3 Caring about privacy

In our exploration of Sicily's mummified denizens, a nuanced ethical dilemma emerged concerning the posthumous privacy rights of these individuals, particularly in relation to the ailments they endured in life (Beckett et al., 2021). Although these individuals—or their families—chose burial within these sacred spaces, divulging the intimate details of their health imposes a layer of vulnerability, potentially conflicting with their desired legacy. To navigate this sensitive terrain, we, alongside the custodians of these remains, concurred that anonymization was imperative in the dissemination of any sensitive findings (Piombino‐Mascali et al., 2021; Squires & Piombino‐Mascali, 2022). By assigning discrete accession numbers to each subject under study, we preserved a thread connecting the biological evidence to individual identities, ensuring the possibility of future reassociation, should ethical considerations permit (Piombino‐Mascali & Beckett, 2021).

This strategy of anonymization was particularly pivotal in a project dedicated to understanding the health, development, and societal roles of juvenile mummies, safeguarding the privacy of these young individuals while facilitating scholarly inquiry. Each mummy was accorded a unique identifier, decoupling distinctive traits from the scientific data published, thereby protecting their anonymity within the public domain (Squires et al., 2024).

A notable deviation from this protocol was the case of Rosalia Lombardo, a child mummified in 1920 and entombed within the Capuchin Catacombs. Her exceptional preservation has not only captivated the public but also positioned her as a focal point of our research efforts (Panzer et al., 2010; Panzer et al., 2013). Given Rosalia's iconic status, anonymizing her data would do little to obscure her identity from the collective memory or academic discourse (Piombino‐Mascali & Zink, 2023). This unique circumstance warranted a departure from our standard practice, openly acknowledging her identity to leverage the attention her case attracts for securing funding dedicated to her preservation. The acquisition of a climate‐controlled display case for Rosalia represents a tangible outcome of this strategy, ensuring her protection from environmental threats and serving as a testament to the delicate balance between ethical research practices and the imperative of conservation (Samadelli et al., 2019).

### 4.4 Mummies and the media

In the ambit of our Sicilian mummy project, a critical consideration was how to ethically and accurately convey our findings to the public. In an era where engaging narratives play a pivotal role in connecting professional research and museum exhibitions with the broader audience, the integrity of the information shared holds paramount importance (Cova [de la], 2023). This is especially true for the numerous crypts across Sicily, many of which rely solely on visitor revenue for their upkeep. It is essential, therefore, that the narratives crafted for public consumption are anchored in authenticity, acknowledge the boundaries of our research, and eschew sensationalism in favor of educational value (Snoddy et al., 2020).

To bridge the gap between scientific inquiry and public interest, our team has incorporated a professional journalist and storyteller since 2009. This collaboration ensures that our engagements with the media—be it through websites, magazines, or both digital and print newspapers—are informed, nuanced, and respectful of the material's sensitivity. This role is crucial not only in crafting content but also in guiding the portrayal of the mummies and the scientific process in a manner that is both accurate and respectful.

To educate and inspire our youngest audience, we published a children's book dedicated to the mummies of Sicily. This book serves as an introduction to the rich heritage of the Capuchin Catacombs, detailing the lives of its most famous residents, the mummification process, and the cultural significance of these remains. Such educational initiatives are vital, particularly as we navigate the challenges of preserving the crypts under adverse environmental conditions and financial constraints (Franco, 2013).

Furthermore, bridging the gap between the scientific community and the public, a trilogy of popular science books has been released. These volumes disseminate current research findings in an accessible format, catering to both professionals and lay readers alike (Piombino‐Mascali, 2009, 2018, 2020).

Moreover, by organizing special events, such as researcher‐led guided tours, we foster a direct dialogue with the public. These tours offer insights into the subtleties of mummification and the historical context of the remains, aspects that may not be fully appreciated without expert guidance. Such interactions not only enhance public understanding but also ensure that the stories of the mummies, and the scientific efforts behind their preservation, are communicated with the depth and respect they deserve (Giallombardo, 2022).

## 5 CONCLUDING REMARKS

In the realm of bioarchaeology, mummies occupy a distinct classification of human remains, identifiable by their preserved features, and as such demand specialized guidelines for their analysis, conservation, and presentation. While recent advancements have introduced ethical protocols for skeletal analysis, these frameworks fall short of comprehensively addressing the unique challenges posed by mummified remains (Gill‐Frerking, 2021). Essential to the integrity of future research is the consideration of cultural nuances, including religious practices and mortuary traditions. The Sicily mummy project exemplifies pioneering efforts in this domain, fostering open dialogue among stakeholders—curators, clergy, scientists, and government officials—thus ensuring the preservation of the mummies' dignity within their sacred contexts (Squires & Piombino‐Mascali, 2022, 2024).

Biological anthropologists play a pivotal role in these investigations, bridging gaps that may exist when incorporating clinical radiologists and pathologists into mummy studies. While these medical professionals offer invaluable insights into the health and potential causes of death of the mummified, their expertise often does not extend to paleoradiology, human osteology, or taphonomy—areas critical to mummy research (Ventura et al., 2022). Hence, incorporating biological anthropologists, who are adept in paleopathology and versed in constructing biological profiles and understanding the biocultural significance of mummies, is imperative. Their guidance is crucial in determining appropriate conservation measures and the safe transportation of these remains. Similarly, the involvement of qualified restorers, who can care for associated artifacts such as coffins and religious items, underscores the necessity of a multidisciplinary approach to the study of Sicily's mummified heritage (Klocke, 2010).

This comprehensive bioarchaeological strategy ensures the safeguarding of mummified remains and the advancement of scientific knowledge, all the while mitigating potential risks to both the remains and the living. Furthermore, the ethical dimensions of mummy research, particularly in the dissemination phase, warrant thorough exploration in academic publications. It is imperative that articles addressing mummified remains delineate the ethical considerations, methodologies, and standards applied, especially in journals not typically focused on historical remains (Squires et al., 2022). The critical review of such articles by biological anthropologists and paleopathologists, who prioritize research driven by specific questions over exploratory studies, can guide the respectful and informed approach to mummy studies, both within Sicily and in broader contexts.

Looking ahead, initiatives by the Sicilian region to recognize cadaver desiccation as part of its intangible cultural heritage (REIS) and to develop precise protocols and recommendations for mummy research signify a commitment to the protection of these unique human remains. Such measures, alongside stringent oversight, are vital for the continued preservation of this fascinating segment of human heritage, which currently faces numerous threats.

## AUTHOR CONTRIBUTIONS

**Dario Piombino‐Mascali:** Conceptualization (equal); data curation (equal); formal analysis (equal); funding acquisition (equal); investigation (equal); methodology (equal); project administration (equal); writing – original draft (lead); writing – review and editing (lead). **Kirsty Squires:** Data curation (equal); formal analysis (equal); funding acquisition (equal); investigation (equal); methodology (equal); project administration (equal); resources (equal); validation (supporting); writing – original draft (supporting); writing – review and editing (supporting). **Albert Zink:** Conceptualization (equal); data curation (equal); formal analysis (equal); funding acquisition (equal); investigation (equal); methodology (equal); resources (equal); supervision (lead); validation (lead); writing – review and editing (supporting).

## Data Availability

All data cited are fully available.
